# Spatial and Temporal Evolutionary Characteristics and Its Influencing Factors of Economic Spatial Polarization in the Yangtze River Delta Region

**DOI:** 10.3390/ijerph19126997

**Published:** 2022-06-07

**Authors:** Yiheng Zhu, Shan Yang, Jinping Lin, Shanggang Yin

**Affiliations:** 1 School of Geography, Nanjing Normal University, Nanjing 210023, China; zyheng537@njnu.edu.cn (Y.Z.); linjinping23@163.com (J.L.); yinshanggang@163.com (S.Y.); 2 Jiangsu Center for Collaborative Innovation in Geographical Information Resource Development and Application, Nanjing 210023, China

**Keywords:** economic development, spatial polarization, spatial and temporal evolution, regional coordination, Yangtze River Delta (YRD) region

## Abstract

Economic spatial polarization is a manifestation of unbalanced urban development. To study the unbalanced development of Chinese cities, this paper selects 41 cities in the Yangtze River Delta (YRD) region, introduces the polarization index and exploratory spatio-temporal analysis to portray their spatio-temporal evolution process, and analyzes the differences in spatial polarization patterns of economic development in three dimensions of economic quantity, quality, and structure. Finally, we use the geographic detector model to explore the driving factors and then propose corresponding policy recommendations. The results show that: (1) the degree of difference in economic development in the YRD region narrowed from 2000 to 2019, and the spatial polarization level of urban economic development showed a fluctuating downward trend, among which the spatial polarization level of the economic structure dimension has been increasing. (2) In terms of spatial distribution, the “Yangtze River Delta urban agglomeration” has economic spatial polarization in the YRD region has become the peak contiguous zone, and the spatial polarization of economic quantity and quality dimensions has formed a “polycentric” pattern, while the spatial polarization of economic structure dimensions shows a stable “one core, multiple sub-center” distribution. (3) From the evolution of spatial polarization, most cities have strong spatial locking characteristics without a transition. Spatially positive polarized are concentrated in the YRD urban agglomeration, and the inter-city neighboring relations are mainly positive synergistic growth, while the negatively polarized cities are mostly distributed in the peripheral areas of the YRD and the neighboring relations are negative synergistic growth. At the same time, the spatially positive polarization effect of the economic quantity dimension and the spatially negative polarization effect of the economic structure dimension among cities are more significant. (4) The economic spatial polarization in the YRD region is mainly dominated by market prosperity and urbanization level, while the driving effect of scientific and technological innovation development on the urban economy has also been expanding in recent years. Promoting the reasonable allocation of marketization, urbanization, and technology among cities with positive and negative spatial polarization in the future will contribute to balanced urban and regional economic development in a coordinated and orderly manner.

## 1. Introduction

Since more than 40 years of reform and opening up in China, after the transition from a planned economy to a market economy, the problem of unbalanced regional development has gradually emerged, and the imbalance of economic development has also transformed from a low level to a high level. Inequality has been widely studied in academia because of its important impact on social cohesion, medical service carrying capacity, and regional sustainable development [1,2,3,4,5,6,7]. The main contradiction in Chinese society has also been transformed into a contradiction between the people’s ever-growing needs for a better life and unbalanced and inadequate development. With the transformation of the principal contradiction and the construction of a well-off society in an all-round way as scheduled, the long-term goal of achieving common prosperity has been put forward for the first time, which confirms that the root cause of the current unequal development of Chinese society is still the imbalance of regional economy [8,9,10].

In the process of regional economic growth, due to the scarcity of resources, it is only possible to give priority to development in areas with better innate endowments and attract resources from surrounding areas to continuously accumulate to them to generate scale effects, and then promote other regions through efficient resource allocation. Therefore, unbalanced development among regions is inevitable [11,12,13,14,15]. In the early stage of the transformation of the economic system, concentrating resources to develop one region will help to achieve the goal of economic growth, form a “polarized” development pole, leading to the continuous widening of the gap, resulting in a “siphon” effect, which affects the development of surrounding areas, especially backward regions. From 1978 to 2019, the average annual growth rate of China’s GDP reached 9.4% [16], but it also faced problems such as increasing social and economic imbalances and environmental unsustainable development [17,18,19,20,21]. To narrow the differences in inter-regional development, the Chinese government has successively implemented the “Western Development Plan”, the “Revitalization of the Northeast”, the “Rise of Central China” and the “Rural Revitalization Strategy” [22,23,24,25,26], and using the remaining economic development capacity of the “polarized” development areas along the eastern coast to stimulate and promote the social development and economic growth of underdeveloped regions such as the northeast, and central and western regions of China [27,28].

Spatial polarization is an important dimension of unequal development, and it has received much attention because it is closely related to the balance of regional development. In the early 1850s, French economist Francois Perroux put forward the concept of the “growth pole” [29], which initially only aimed at the problem of industrial development. Subsequently, Swedish economist Myrdal proposed the “Circular Cumulative Causality Theory”, which enriched the concept of growth poles [30], arguing that the process of economic development does not occur at the same time and spreads evenly in space, but starts from areas with better original endowments and relies on its advantages to achieve advanced development. The “center-periphery theory” proposed by American economist Friedmann in 1966 combines growth pole theory and geospatial theory organically. The regions that take the lead in development for a variety of reasons become the “center”, while other regions become the “periphery” because of their slow development [31], and there is an unbalanced relationship between them, in which the “periphery” depends on the development of the “center”. The “spatial polarization” studied in this paper is the state or result of deviation from the global average horizontal differentiation distribution caused by the uneven agglomeration of economic factors in space to a certain degree, and the degree of deviation is called the “spatial polarization degree”.

The research on spatial polarization in academia is mainly divided into two aspects: theoretical and empirical. In theoretical research, since the 1990s, Esteban and Ray proposed the Esteban–Ray exponential model (ER index) through the conceptual reasoning of “class” [32]; Wolfson, based on the Lorenz Curve, proposed the Wolfson index (W index) to measure the polarization of income and wealth distribution [33]; Kai-yuen Tsui and You-qiang Wang derived the Tsui–Wang index (TW-index) based on the Wolfson index using the sorting axiom [34], etc., which greatly enriches the problem of using mathematical models to quantitatively measure the degree of polarization in regional development in the study of spatial polarization theory. In terms of empirical research, some scholars have carried out economic development extremes in the United States, the United Kingdom, Canada, the three northeastern provinces of China, Jiangsu Province, Guangdong province, and other regions by using relevant measurement methods to take the GDP or per capita GDP as a single indicator. It also determines a variety of explanation mechanisms for spatial polarization, such as factor flow, urban prejudice, agglomeration economy, and knowledge spillover [35,36,37,38,39,40,41]. Other scholars study the spatial polarization of Japanese land price evolution and Ukrainian public opinion polls with the help of the spatial variation function model, micro-dynamic model, and exploratory spatial analysis [42,43]. Additionally, some scholars measure and explore the level of spatial polarization in the development of urbanization, railway transportation, and scientific and technological innovation, and have found that the regional labor market, economic structure, settlement structure, population density, transportation infrastructure, etc., all affect the development of regional imbalances from different aspects [44,45,46,47,48,49,50,51,52,53].

Looking at the existing research, current scholars have explored and analyzed the phenomenon of regional development inequality and spatial polarization of factors from various aspects, which has laid a good research foundation. However, the following issues still need to be further explored. First, most of the existing research is carried out on a single factor or a single dimension. As China’s economic development enters a new normal, the impact of economic factors on regional balanced development is diverse and complex, and economic quantity, quality, and structure all play a role in China’s economic growth to varying degrees. Multi-dimensional analysis of the differences brought about by economic factors in the process of regional spatial polarization is of great significance for promoting sustainable economic development and social stability. Second, the static spatial pattern of economic development has been studied, but the differences and impacts brought about by the dynamic changes in the local structure of economic spatial polarization need to be further decomposed and explored. Therefore, this paper takes cities above the prefecture level (41 in total) in the YRD region of China as an example to construct an evaluation index system for the level of economic spatial polarization. From the perspective of spatio-temporal dynamic evolution, this paper combines the phenomenon of spatial polarization with the promotion of regional coordinated development and aims to study the process, characteristics, and influencing factors of economic spatial polarization in different dimensions in the YRD region from 2000 to 2019 to provide a scientific basis for regional coordinated development.

The remaining part of this paper is organized as follows: Section 2 explains the methodology and data; Section 3 analyzes the temporal and spatial evolution process and characteristics of the spatial polarization of economic development; Section 4 explores the factors that affect the spatial polarization of economic development; Section 5 discusses empirical evidence and makes relevant policy recommendations; and Section 6 presents conclusions.

## 2. Materials and Methods

### 2.1. Overview of the Study Area

**Figure 1:**
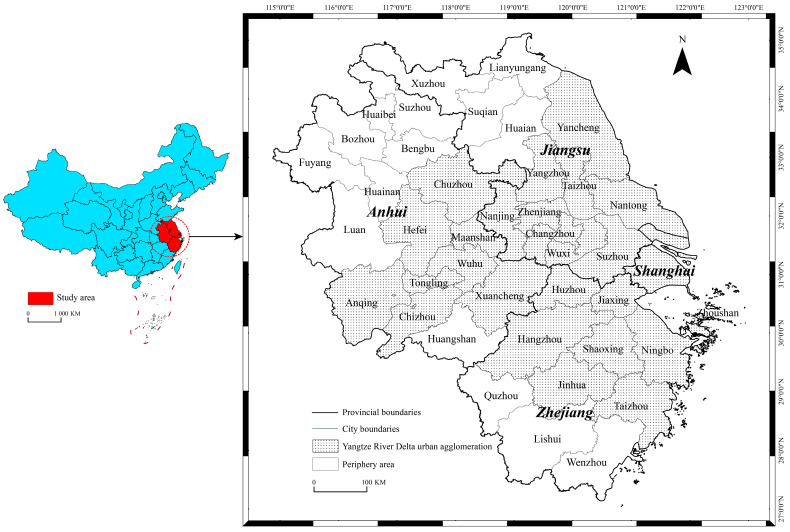
Study area.

The Yangtze River Delta (YRD) region is located in the lower reaches of the Yangtze River in China, close to the Yellow Sea and the East China Sea, and is located at the intersection of the river and the sea. According to the outline of the Plan for Regional Integrated Development of the Yangtze River Delta issued in December 2019, there are a total of 41 cities at the prefecture-level and above in Shanghai, Jiangsu, Zhejiang, and Anhui Province. The YRD region is an important intersection between China’s “Belt and Road Initiative” and the Yangtze River Economic Belt. It is an important engine for China’s economic and social development and an important platform for participating in international competition. As of the end of 2019, the total land area of the YRD region was 358,000 square kilometers, the regional GDP reached CNY 23.75 trillion, accounting for about 23.9% of the country, and the urbanization rate of the permanent population was 67.23%, 6.63 percentage points higher than the national average [16], making it one of the regions with the strongest comprehensive strength and the most developed economy in China (Figure 1).

### 2.2. Index System and Data

Economic development has rich connotations, covering many aspects of economic growth, and can better meet the economic development model, structure, and dynamic state of the people’s growing real needs [54]. The quantity of economic development is related to the production and life of the people, maintaining a certain speed and scale of economic growth is a necessary supporting condition for urban economic development. The quality of economic development is an objective reflection of labor productivity and market consumption level and has a certain impact on the stable development of society. The economic structure reflects the advanced level of industrial sophistication and urban competitiveness and provides a basis for the improvement of urban economic strength and anti-risk capabilities. The coordinated and orderly development of the three is of great significance [55]. Based on this, six indicators are selected from the three dimensions of economic quantity, economic quality, and economic structure to construct the spatial polarization level evaluation system of economic development in the YRD region. The specific indicators and meanings are shown in Table 1.

The research data mainly come from the Statistical Yearbook of Chinese cities from 2001 to 2020, the Statistical Yearbook of Shanghai, the Statistical Yearbook of Jiangsu Province, the Statistical Yearbook of Zhejiang Province, the Statistical Yearbook of Anhui Province, the statistical yearbooks of prefecture-level cities and the statistical bulletins of national economic and social development of prefecture-level cities. Taking into account the adjustment of administrative divisions and the continuity of data, the data were divided and merged according to the latest administrative divisions during the study period, and the data of some missing years were supplemented by interpolation.

### 2.3. Methods

#### 2.3.1. Entropy Weight Method

Entropy originally originated from the concept of thermodynamics in physics, which mainly reflects the degree of chaos in the system. It has been widely used in research fields such as economic development evaluation and urban development quality [56,57]. Entropy mainly characterizes the discrete degree of the index. The smaller the entropy value is, the greater the discrete degree of the index is, and the greater the weight of the index for the comprehensive evaluation, otherwise the smaller the weight of the index for the comprehensive evaluation. Using the entropy method to determine the index weight can not only overcome the problems of randomness and assumption that cannot be avoided by the subjective weighting method but can also effectively solve the problem of overlapping information among multiple index variables. Therefore, this paper uses information entropy to determine the index weight and uses the comprehensive weighting method to obtain the economic agglomeration index of the YRD region from 2000 to 2019 for evaluation. For detailed calculation steps, see Reference [58].

#### 2.3.2. Spatial Polarization Measurement Model

The Tsui–Wang index (TW-index) and the Coefficient of Variation (*CV*) are used to comprehensively measure the process and evolution characteristics of the economic spatial polarization in the YRD region in the time series and spatial dimensions and explore the impact of the flow of factors between cities in the study area on the spatial polarization. First, the TW-index is used to measure the degree of regional spatial polarization, and the calculation formula is as follows [59,60],

(1) $$TW=\frac{\theta }{L}{\sum_{i=1}^{n}\pi_{i}\left|\frac{S_{i}-m}{m}\right|}^{r}$$

where *L* is the flow of all urban population factors, *π_i_* is the flow of population factors of city *i*, *n* is the number of cities in the region, *S_i_* is the economic agglomeration index of city *i*, *m* is the median of the economic agglomeration index of all cities, *θ* is a positive constant scalar, and *r* ∈ (0, 1). In this paper, we assume that *θ* = 0.5, *r* = 0.5.

Secondly, the *CV* was introduced to analyze the variation characteristics of regional spatial polarization on the longitudinal time scale at different scales. The larger the *CV* value, the greater the difference in spatial polarization in the study area, and the smaller the difference on the contrary. The expression for its coefficient of variation is [61],

(2) $$CV=\sqrt{\overset{n}{\underset{i=1}{\sum }}\frac{{\left(S^{i}-\overset{ˉ}{S}\right)}^{2}}{\left(n-1\right)}}/\overset{ˉ}{S}$$

where *CV* is the coefficient of variation, *n* is the number of cities in the YRD, and *S* is the average value of the economic agglomeration index of all cities in the Yangtze River Delta region.

Finally, the economic agglomeration index is used to measure the spatial polarization (*P*) of each city. The calculation formula is as follows,

(3) $$P=S^{i}/\overset{ˉ}{S}$$

where *P* is the spatial polarization; if the *P* value is greater than 1, it means that the spatial polarization level of the city is higher than the regional average level, resulting in a positive polarization phenomenon, which is called a positive polarization city. Otherwise, it is called a negative polarization city.

#### 2.3.3. LISA Time Path

The LISA (Local Spatial Autocorrelation) temporal path is a continuous expression of the positional shift of a spatial unit in Moran’s *I* scatterplot [62]. Visualizing the paired movement of the economic polarization degree of spatial units and their spatial lag terms explains the spatio-temporal co-evolution of economic polarization and reflects the local spatial differences and the spatio-temporal dynamics of economic polarization changes [63]. The main indicators of the LISA time path include relative length, curvature, direction, etc., which reflect the dynamic characteristics, volatility characteristics, and integration characteristics of urban local spatial structure, respectively, and the relevant expressions are as follows [64],

(4) $$d_{i}=\frac{n\sum_{t=1}^{T-1}d\left(L^{i,t},L^{i,t+1}\right)}{\sum_{i=1}^{n}\overset{T-1}{\underset{t=1}{\sum }}d\left(L^{i,t},L^{i,t+1}\right)}$$

(5) $${ℰ}_{i}=\frac{\sum_{t=1}^{T-1}d\left(L^{i,t},L^{i,t+1}\right)}{d\left(L^{i,t},L^{i,T}\right)}$$

(6) $$\theta^{i}=\arctan\frac{\sum_{j}\sin\theta^{j}}{\sum_{j}\cos\theta^{j}}$$

where *d_i_*, *ε_i_* and *θ_i_* are the relative length, curvature, and average moving direction of city *i*, respectively, *n* is the total number of urban units, *T* is the annual time interval, *L_i_*, *t* is the LISA coordinate of city *i* in time *t*, and *d(L_i_*_,*t*_*, L_i_*_,*t*+1_*)* is the moving distance of city *i* from time *t* to *t* + 1.

#### 2.3.4. LISA Space–Time Transition

Based on local spatial autocorrelation, the moving distance, direction, agglomeration and other attributes of each spatial unit in Moran’s *I* scatter-gram within a specific time interval are embedded into the traditional Markov chain, and the local Markov transfer and space–time transition are proposed. The transition is used to decompose and reveal the spatial dependence of geographical phenomena and is divided into four types: Type1, Type2, Type3 and Type0 [65]. Type0 indicates that the transition between the city itself and its neighborhood does not occur with time; Type1 means that the city itself transitions, but the neighborhood remains unchanged; Type2 means that the city itself remains unchanged, but the neighborhood transitions; Type3 means that both the city itself and its neighborhood have transitioned. According to the transition direction, Type3 can be divided into two types: Type3A and Type3B, in which the former indicates that the transition direction of the city itself is the same as that of the neighborhood, while the latter indicates that the transition direction of the two is opposite. Rey defined the space-time flow (*SF*) and space–time condensation (*SC*) in the regional system to represent the spatial pattern path dependence and locking characteristics of the research object [66]. The ratio of the number of transitions of a certain type to the total number of transitions (*m*) that may exist in the system during the study period,

(7) $$SF=\frac{T_{1}+T_{2}}{m},\;SC=\frac{T_{0}+T_{3A}}{m}$$

where *T*_0_, *T*_1,_ *T*_2_ and *T*_3A_ is the transition numbers of Type0, Type1, Type2 and Type3A, respectively; *m* is the total number of transitions.

#### 2.3.5. Geographic Detector

With the help of the geographic detector model, the influencing factors of regional spatial polarization are explored and analyzed. The geographic detector is mainly composed of four parts: risk detector, factor detector, ecological detector, and interaction detector. This study mainly focuses on the factor detection and analysis part, which is mainly used to analyze the degree of interpretation of the influence factors on the research object. The geographic detector values of the influence factors can be expressed as [67,68],

(8) $$q=1-\frac{1}{n\sigma^{2}}\sum_{i=1}^{K}n_{i}\sigma_{i}^{2}$$

where *nσ*^2^ represents the total variance of the agglomeration degree of urban elements in the region, $n_{i}\sigma_{i}^{2}$ represents the variance of the area within the layer, *n* is the number of cities in the entire region, and *K* is the number of areas in the layer. The larger the *q* value, the greater the explanatory power of the geographic detection factor in the spatial polarization of the affected area, and the smaller the explanatory power of the geographic detection factor in causing the spatial polarization of the affected area.

## 3. The Spatio-Temporal Characteristics of Economic Spatial Polarization

### 3.1. Temporal Variation of Economic Spatial Polarization

**Figure 2:**
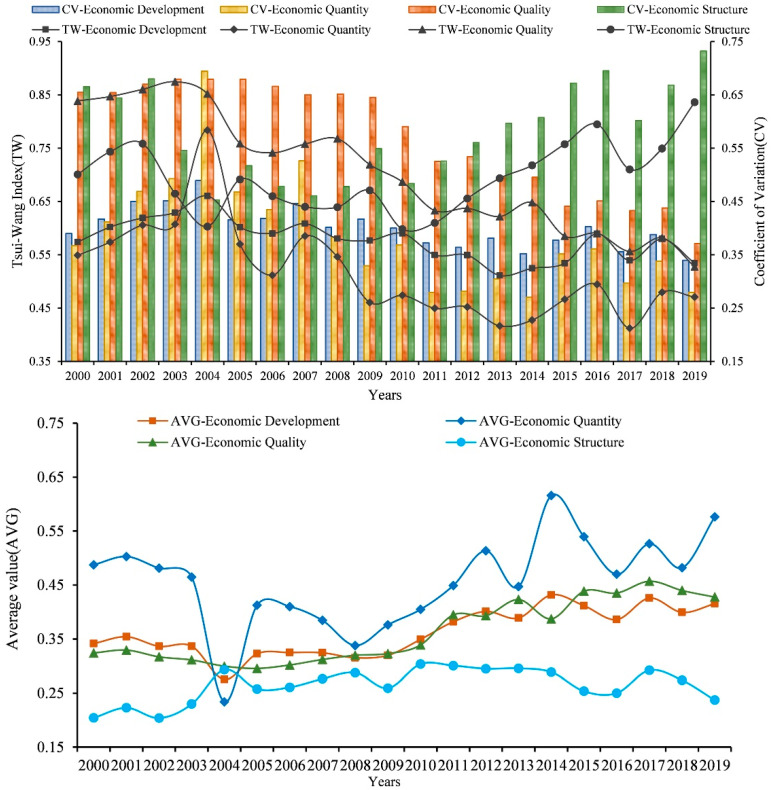
Economic development and evolution of TW index, CV and average value of each dimension.

The *CV* of economic development and the TW-index reflect the overall spatial polarization level of economic factors in the YRD region. The relationship between economic quantity, economic quality, and economic structure over time can further reveal the development trend of spatial polarization within economic factors and have a deeper understanding of the role of each dimension in the process of economic spatial polarization. Therefore, the *CV* and the TW-index were introduced to measure the economic development and the spatial polarization differences and evolution characteristics of each dimension in the YRD region from 2000 to 2019 and analyze it combined with the average level change of economic development (Figure 2).

According to the evolution process of the long-term TW-index in the YRD region and the *CV*, the TW-index and the *CV* of economic development dropped from 0.574 and 0.389 in 2000 to 0.534 and 0.339 in 2019, respectively; the former decreased by 6.9%, and the latter decreased by 12.9%. The decrease in the two indices indicates that the level of regional spatial polarization and the degree of difference are both decreasing, indicating that based on the continuous improvement of the economic development level in the YRD region in the past 20 years, the TW-index of inter-city economic development has decreased, the difference in economic development is in an obvious narrowing trend, and the inequality in regional economic development has been alleviated to a certain extent.

The economic elements are further decomposed into three dimensions, economic quantity, economic quality and economic structure, to analyze the evolution among the three. From 2000 to 2019, the *CV* of economic quantity and economic quality and the TW-index showed a fluctuating downward trend. The TW-index and *CV* of economic quantity dropped from 0.549 and 0.367 in 2000 to 0.470 and 0.279 at the end of the study period; both decreased by 14.4% and 23.9% in turn. The TW-index and *CV* of economic quality decreased from 0.839 and 0.655 at the beginning of the study period to 0.527 and 0.370 at the end of the study period, and the two decreased by 37.1% and 43.4%, respectively, indicating that the quantity and quality of economic development in cities in the YRD region have a trend of coordinated development, and the difference in economic quantity and quality between cities is gradually zoomed out. The evolution of the economic structure is different from that of the other two dimensions. At the end of the research period, compared with the beginning of the research period, the TW-index and the *CV* increased from 0.700 and 0.665 in 2000 to 0.836 and 0.732 in 2019, respectively. It has increased by 19.3% and 10.1%, indicating that the economic structure of the YRD region is in a situation of differentiated development, the development inequality between cities has increased, and the level of spatial polarization has been continuously increasing, which has an impact on the process of coordinated development of economic elements among regions. From the decomposition of the process of economic spatial polarization in the past 20 years, before 2012, except for the sudden rise and fall of the TW-index of economic quantity and economic structure in 2004, the TW-index of economic quality was always higher than that of economic structure and economic quantity. Since 2012, the continuous fluctuation of the TW-index of economic structure has exceeded that of economic quality, forming a spatially polarized evolution trend of economic structure > economic quality > economic quantity.

In addition, combining the average changes of economic development and its three dimensions of quantity, quality and structure, it can be seen that the average level of eco-nomic development has fluctuated and increased in the past 20 years, and the average value of economic development and economic quantity dimensions has dropped sharply in 2004. Among them, since 2000, the average level of economic development has in-creased by 21.65%, and the three dimensions of economic quantity, economic quality and economic structure have increased by 18.24%, 31.97% and 16.02%, respectively, and the growth rate of economic quality is particularly obvious. Data analysis shows that the convergence of the shrinking differences in the spatial polarization of economic development in the YRD region is the result of high-quality coordinated development, rather than “low-level coordination” formed at the expense of lower economic growth rates.

### 3.2. Spatial Differentiation of Economic Spatial Polarization

**Figure 3:**
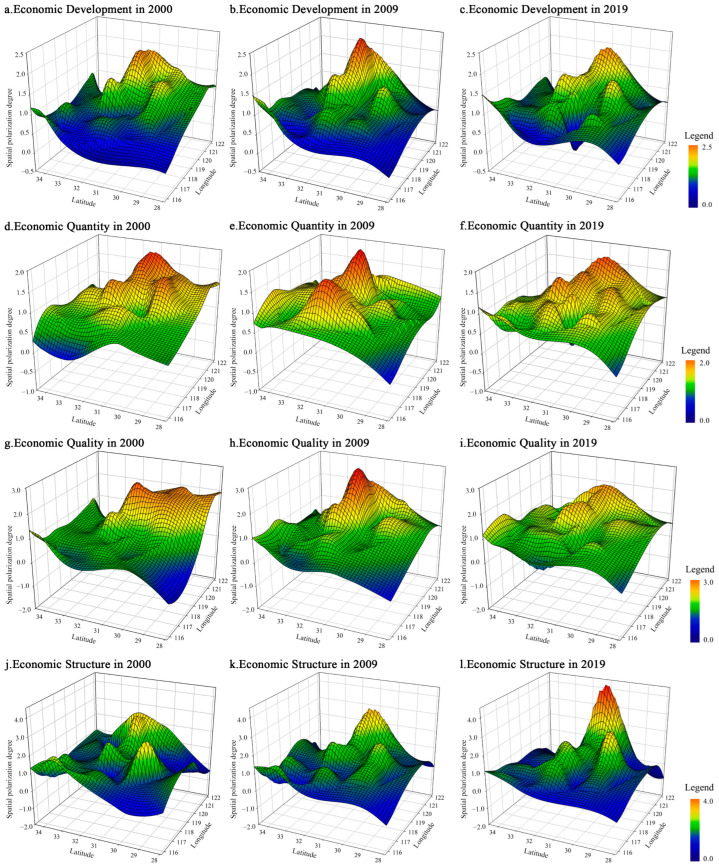
The Evolution Pattern of Economic Spatial Polarization in the YRD Region.

Based on the spatial polarization of the three dimensions of economic quantity, economic quality, and economic structure in the evolution of economic spatial polarization in the YRD region from 2000 to 2019, using SigmaPlot10.0, three typical years of 2000, 2009, and 2019 were selected for spatial visualization, and the economic development in three years and the evolution trend of spatial polarization in three dimensions were obtained (Figure 3).

The spatial polarization distribution of economic development (Figure 3a–c), generally shows a core-peripheral structure of “high in the middle and low in the north and south”. The level of spatial polarization decreases along the Yangtze River axis from Shanghai-Nanjing as the center to Anhui, northern Jiangsu, and southwestern Zhejiang. With time, the area of “polarization bulge” in the YRD region is increasing, and the phenomenon of spatial polarization is obvious, but the level of polarization has declined. The main manifestation is that the peak area of economic spatial polarization has gradually evolved into a polarized continuous area of “urban agglomeration” from the cities along “Shanghai-Nanjing-Hangzhou-Ningbo” from 2000 to 2019, and the peak value of economic spatial polarization has decreased by 0.047. While the degree of spatial polarization has declined, the polarized area has continued to expand and is mainly concentrated in the urban agglomeration of the YRD with Shanghai as the center. At the same time, it effectively drives the economic growth of the surrounding areas and makes a certain contribution to the coordinated and sustainable development of the region.

Comparative analysis of the spatial differentiation among the three economic dimensions shows that the spatial polarization evolution of economic quantity (Figure 3d–f) and economic quality (Figure 3g–i) are similar and convergent. The “single-center” structure of spatial polarization in 2000 evolved to the “multi-center” polarization pattern at the end of the study period. Specifically, the spatial polarization peak city of economic quantity, Shanghai, evolved from 1.85 in 2000 to 1.61 in 2019, and the polarization degree dropped by 12.97%, forming a new polycentric pattern with Nanjing, Suzhou, Wuxi, Hangzhou, and Hefei. The spatial polarization peak of economic quality in Wuxi evolved from 2.34 in 2000 to 1.54 in 2019, forming a new multi-center pattern with Shanghai, Ningbo, and southern Jiangsu. The spatial polarization of economic structure (Figure 3j–l) is opposite to the evolution trend of the other two dimensions. The phenomenon of spatial polarization is obvious, and the degree of polarization is deepening, and it has a stable “one-core, multiple sub-center” structure, mainly manifested in the evolution of core Shanghai from 2.47 at the beginning of the research period to 4.22 at the end of the research period, much higher than other cities in the YRD region. Additionally, the sub-center evolved from Nanjing, Suzhou, Huangshan, and Zhoushan in 2000 to Nanjing, Suzhou, Wuxi, and Hangzhou, and the spatial polarization of the core and sub-center cities are still intensifying.

Combining the different types of positive and negative evolution of economic spatial polarization, the cities are classified according to the differences in the types of spatial polarization between the beginning and the end of the study period. The cities with positive or negative polarization types in the beginning and end years are “positive–positive” and “negative–negative”, respectively, and the cities with negative polarization at the end of positive polarization in the initial year are “positive–negative”; otherwise, it is a “negative–positive”-type city.

**Figure 4:**
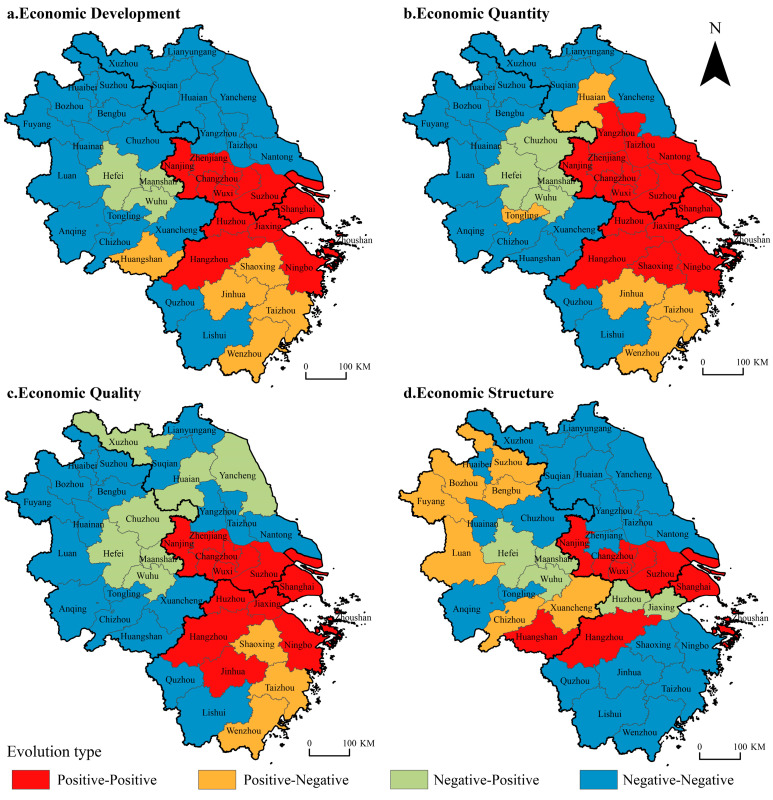
Evolution types of economic spatial polarization in the YRD.

In the evolution of economic spatial polarization (Figure 4), there are 11 positive–positive cities, accounting for 26.8% of all cities in the YRD, and they are all concentrated in the YRD urban agglomeration. The negative–negative cities are mainly concentrated in northern Jiangsu, western Zhejiang, and northern and southern Anhui, mostly in the peripheral areas with a less developed economy in the YRD, accounting for 53.7% of all cities. The positive–negative cities are distributed in central and southern Zhejiang and Huangshan, and their economic development in 2019 is slower than that in 2000. The negative–positive cities are concentrated in Hefei, Wuhu, and Ma’anshan in central Anhui, indicating that the economic development growth level of these three cities is more obvious compared to the beginning of the study period.

The evolution type of spatial polarization is analyzed in three dimensions. In the past 20 years, there were 15 cities in the “positive–positive” type of evolution in terms of economic quantity, 12 in economic quality, and 8 in economic structure; the number decreased in turn, except Huangshan in Anhui Province. In the economic structure, other “positive–positive” cities are concentrated in the YRD urban agglomeration. Among the “negative–negative” evolution cities, there are 17, 19, and 21 cities in terms of economic quantity, quality, and structure, respectively. The cities in the first two dimensions are concentrated in northern Jiangsu, western Zhejiang, and northern and southern Anhui. The economic structure dimension is mainly concentrated in northern Jiangsu and southern Zhejiang and scattered in Anhui Province. The “positive–negative” cities in the dimensions of economic quantity and quality are concentrated in the central and southern parts of Zhejiang. The former is also distributed in Huai’an in Jiangsu province and Tongling in Anhui province, while the economic structure dimension is only distributed in the northern and southern regions of Anhui. From the perspective of the “negative–positive” evolution distribution, except that the three dimensions are relatively concentrated in central Anhui, the economic quality is also scattered in northern Jiangsu and the economic structure is scattered in northern Zhejiang.

From the evolution and distribution of the economic spatial polarization from 2000 to 2019, it can be seen that the cities with positive economic development are mainly concentrated in the YRD urban agglomeration, and the cities in central Anhui have achieved remarkable economic development in recent years. The negatively polarized cities are mostly distributed in the underdeveloped areas on the periphery of YRD, forming an obvious “urban agglomeration-periphery area” spatial structure of positive and negative polarization. The “positive–positive” cities in the three dimensions of economic quantity, quality, and structure are decreasing in turn, while the “negative–negative” cities are increasing in turn, indicating that the spatial positive polarization effect of economic quantity and the spatial negative polarization effect of the economic structure is more significant.

### 3.3. LISA Time Path

**Figure 5:**
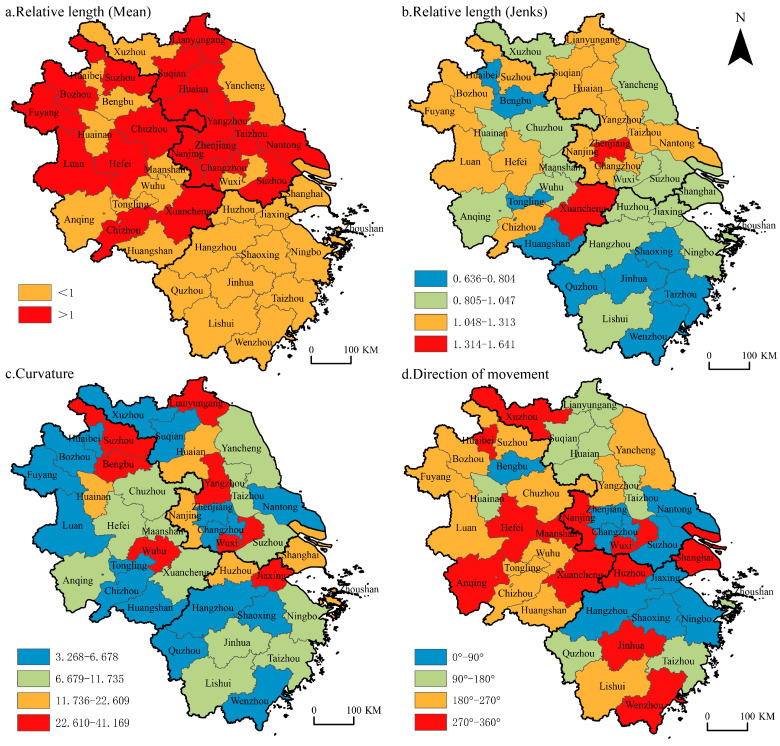
Relative length, curvature, and moving direction of LISA time path.

The relative length of the LISA time path reflects the dynamic characteristics of the local spatial structure of spatial polarization in the economic development of cities in the YRD region. During the study period, if the urban movement length exceeds the average value of all cities, the relative length is greater than 1; otherwise, it is less than 1. A visualization is shown in Figure 5a. There are 18 cities with a relative length above the average in the YRD, accounting for 43.9% of the total. Additionally, they are mainly distributed along the Yangtze River and in northern Jiangsu and northern Anhui, with relatively dynamic local spatial structures. There are 23 cities whose relative length is lower than the average, accounting for 56.1%. They are mainly distributed in Zhejiang province and scattered in southern Anhui and southern Jiangsu, with a relatively stable local spatial structure. This result is consistent with the evolution characteristics of the spatial polarization pattern of overall economic development. According to the classification method of natural breaking points, the relative lengths are divided into four categories (Figure 5b). According to the average value of the urban agglomeration and its surrounding areas, the urban agglomeration in the YRD (1.02) is more than that of the periphery of the YRD agglomeration (0.98), indicating that the local spatial structure of the peripheral areas of urban agglomeration is more stable than that of urban agglomerations.

According to the results of the curvature of the economic spatial polarization in the YRD region, its values are all greater than 1, indicating that the spatial polarization of regional economic development has obvious migration characteristics. The economic polarization curvature of 41 cities is classified by ArcGIS’s natural discontinuity point classification method, and the division result is visualized as shown in Figure 5c. Cities with a high curvature are scattered, such as Bengbu, Suzhou, Lianyungang, Zhoushan, etc. Most of these cities are located on the periphery of provinces. The economic development of these cities is limited by the core area and has a large fluctuation in the direction of spatial dependence. In terms of the average value of the urban agglomeration and its surrounding areas, the YRD urban agglomeration (12.67) is more than that of the periphery of the YRD agglomeration (12.15), indicating that the urban agglomeration has a more fluctuating spatial dependence compared with the peripheral areas of the agglomeration.

The visualization results of the moving direction of the economic spatial polarization in the YRD region are shown in Figure 5d. In total, 21 cities showed synergistic growth. During the study period, these accounted for 51.2% of the total, indicating that the evolution of the spatial polarization pattern of economic development in the YRD region has a certain degree of evolution. Among them, 42.9% of the cities in southern Jiangsu and northeastern Zhejiang showed positive synergistic growth characteristics, 57.1% of cities in northern Anhui, southern Anhui, central Jiangsu, and Zhejiang Lishui showed a negative synergistic growth trend, while other cities such as Shanghai and Nanjing, Wuxi, Hefei, Ma’anshan, etc., show the opposite direction of transition from neighboring cities.

### 3.4. Spatio-Temporal Transition Analysis of LISA

The LISA time path reveals the changing trend of each city in the Moran scatter chart and then explores the transfer characteristics and evolution trend of the local spatial correlation types of the economic spatial polarization in the YRD with the help of the probability transfer matrix and spatio-temporal transition proposed by Rey.

As shown in Table 2, from 2000 to 2019, the most common type of transition in the YRD is Type0, which means that there is no quadrant transition between the city itself and its neighborhood over time, and the probability of this type of transition is 80.8%, indicating that there is certain transfer inertia in the spatial polarization of regional economic development, the current type of economic polarization in each city is difficult to change, and the spatial polarization pattern of economic development remains relatively stable. During the study period, Type1 and Type2 indicate that there is only one transition between the city itself and the neighborhood, with a probability of 9.1% and 6.1%, respectively, indicating that there is a possibility of transfer between the local spatio-temporal correlation categories of the economic spatial polarization in the YRD. Among them, the probability of *LH_t_*→*LL_t_*_+1_ and *HL_t_*→*LL_t_*_+1_ transition is about 20%, and the probability of *LH_t_*→*HH_t_*_+1_ and *HL_t_*→*HH_t+_*_1_ transition is about 30%, indicating that the trend of high-value clustering is more significant than that of low-value clustering. Type3 refers to the transition of the city itself and its neighborhood, with the lowest probability of 4.0%, indicating that the probability of spatial polarization jump transfer of economic development in the YRD is low. The space–time flow (*SF*) and space–time condensation (*SC*) is calculated from the number of transition types. The space–time condensation is as high as 82.4%, and the space–time flow is only 15.2%, which further indicates that the economic spatial polarization in the YRD region has strong transfer inertia, and the spatial pattern of each city is relatively stable, showing high path-locking characteristics.

## 4. Analysis of Influencing Factors of Economic Spatial Polarization

### 4.1. Selection of Influencing Factors

The growth and development of the urban economy are affected by many factors such as labor force, industry, capital, and technology. Combined with the research practice of scholars on economic development, major dimensions such as urbanization, industrialization, science technicalization, marketization, accessibility, and openness are selected as representatives. The construction of urbanization can promote various production factors to gather in cities and promote the transformation and upgrading of urban industrial structure. Industrialization can improve social productivity and enrich people’s material life, marketization reflects people’s consumption level and purchasing power, and a good economic development environment provides a strong guarantee for consumption growth. Transportation, as an important urban infrastructure, is a prerequisite for economic development, plays an important role in shortening the distance between cities, attracting domestic and foreign investment and is conducive to the effective flow and rational allocation of capital. Technological development is conducive to the reform of productivity, production efficiency, and production methods and promotes the transformation of economic development level to a higher level. Openness to the outside world is conducive to the development of foreign trade and the upgrading of industrial structure and, to a certain extent, promotes the development of secondary and tertiary industries. Based on this, six characteristic variables were selected to detect the impact on the economic spatial polarization in the YRD region (Table 3).

### 4.2. Factor Detection Results

Using the factor detection part of the geographic detector, we explored the influence of each characteristic factor on the overall economy spatial polarization in 2000, 2009, and 2019 (Table 4).

The ranking of the influencing factors of economic spatial polarization in the YRD region in different periods is different, and the overall influence coefficient is relatively high. Market prosperity and urbanization level both rank among the top three, while openness and transportation convenience have relatively little impact on economic polarization, and in recent years, the driving effect of scientific and technological innovation has gradually increased. Specifically, according to the detection results of different years, the top three factors affecting the economic spatial polarization in 2000 are market prosperity, industrialization level, and urbanization level, and the impact coefficients are all higher than 0.30. The influence coefficients of market prosperity rank first with 0.555, and the industrialization level and urbanization level are 0.492 and 0.310, respectively. In 2009, the economic spatial polarization was still most affected by market prosperity, and the influence coefficient was higher than that in 2000, which was 0.675. The urbanization level of transportation convenience ranked second and third, respectively. By 2019, the economic spatial polarization in the YRD region is most affected by the urbanization level, technological level, and market prosperity, with influence coefficients of 0.697, 0.589, and 0.477, respectively.

Further comparative analysis of the changes in influencing factors in the YRD region in different periods shows that the two factors of marketization and urbanization have always run through the evolution of the economic spatial polarization pattern since 2000, while the influence of industrialization level is gradually weakening. It shows that, in the early stage, because the YRD region has the geographical advantage of being located on the east coast of China, it has priority to development driven by its source endowment conditions and promoted the growth of the urban economy, which has brought prosperous market trade demand, transaction volume and primitive economic accumulation to the region, which has driven the development of urban industry and made it the most developed economic zone in China. The transportation convenience factor presents an inverted “U”-shaped development trend from weak to strong and then weak, indicating that, with the development of urbanization, as the main area of Chinese urbanization, the agglomeration of the population from different regions and cities to the YRD is obvious. The improvement of the transportation network in some developed cities from 2000 to 2009 has driven the development of the regional economy. However, based on the abundant labor force and the overall construction of transport infrastructure brought about by the population dividend, the dividend effect of convenient transportation is gradually weakened, and the spatial polarization impact of accessibility on urban economic development is also reduced. In the guiding opinions on “Further Promoting Reform and Opening Up and Economic and Social Development in the Yangtze River Delta Region” in September 2008, the State Council proposed to speed up the optimization and upgrading of the industrial structure, and the influence of the science and technology has been gradually increased. It has become the second-largest influence factor in 2019, indicating that scientific and technological innovation plays a stronger role in stimulating economic vitality and promoting economic development in the Yangtze River Delta region. In addition, the influence level of the openness degree to the outside world has always been in the middle and lower reaches of the six major factors in the past 20 years, indicating that the economic spatial polarization in the YRD region is less affected by foreign investment, and the spatial polarization effect is also gradually reducing.

## 5. Discussion

Taking the spatiotemporal differentiation and correlation characteristics of spatial polarization of urban multi-dimensional economic development as the breakthrough point, this paper adopts the analysis method of time path and spatiotemporal transition to analyze the partial development of economic inequality in 41 prefecture-level cities in the YRD in the past 20 years. The dynamics, direction of dependence, spatial integration and its influencing factors of spatial structure changes are studied, which is a further en-richment and supplement to the previous studies that focus more on spatial patterns and ignore the dynamic changes of local structures.

From the research and analysis results, the discovery of spatial polarization of eco-nomic development in the YRD region is basically consistent with the results of related studies in Jiangsu Province [11] and Guangdong Province [40]. Therefore, our research provides strong evidence for the existence and even aggravation of the core–periphery structure and provides strong evidence for the research on spatial polarization in China, which exists not only in the YRD region, but is common in most provinces and urban agglomerations in China. Spatial polarization has become the most important dimension of unbalanced development among regions at different scales in China, and it must attract more attention from policymakers. In addition, the research found that the economic development level of the YRD region has indeed improved significantly since 2000, which has certain advantages and is in the forefront compared with other parts of China. However, while the inter-regional inequality of economic quantity and quality has been alleviated, the regional unequal development phenomenon of the economic structure has intensified, and the regional inequality problem in the YRD region is more complicated than ever, which is not conducive to the sustainable development of the economy and society.

As China’s economic development has entered a new normal, more attention has been paid to the economic development model of structural adjustment and stable growth, which has brought new opportunities for the healthy and sustainable economic development of the YRD region. In this context, policy suggestions for optimizing the economic development of the YRD region are put forward: on the one hand, we should continue to promote the economic development of space-positive regions to drive the common development of surrounding space-negative cities and reduce the siphoning effect of space-positive cities on space-negative cities. The policy strength of spatially positive urban structure adjustment and transformation and upgrading should be increased, full play should be given to the comparative advantages of each city, and new growth poles should be constructed to achieve a win–win situation of spatial agglomeration and coordinated development. On the other hand, it is necessary to construct a new urban cooperation model of “cross-regional counterpart assistance” that extends and promotes the exploration experience of the north–south joint construction of parks in Jiangsu province, strengthens the optimal allocation of spatially positive urban resources on a larger scale, and provides new support points for regional economic growth. At the same time, it is necessary to strengthen infrastructure construction, facilitate the circulation of factors, expand the scope of the space-positive city’s driving effect on surrounding areas, and promote the coordination and sustainability of regional economic development.

## 6. Conclusions

As an important engine of China’s economic and social development, the YRD region is crucial to achieving coordinated and sustainable development by continuously narrowing regional gaps. Based on the measurement of the spatial-temporal evolution pattern of economic spatial polarization in the YRD region from 2000 to 2019, this paper analyzes its temporal-spatial dynamic characteristics through LISA time paths and temporal-spatial transitions and uses the geographic detector model to detect its influencing factors, and draws the following main conclusions:

(1) The economic spatial polarization shows a downward trend of fluctuation over time, and the development difference between cities gradually narrows. In terms of spatial distribution, the polarization peak zone is gradually expanded and evolved from cities along the “Shanghai-Nanjing-Hangzhou-Ningbo” to the polarized continuous area of “urban agglomeration”, with a gradient from the peak area as the center to the periphery, and the inequality of economic development in the YRD region transitions from low level to high level.
(2) There are obvious differences in the spatial polarization of different economic dimensions. From 2000 to 2019, the economic quantity and economic quality show a fluctuating downward trend, and the economic structure is in the process of fluctuating and rising. In space, the economic quantity and quality pattern have evolved from “single-center” to “multi-center”, while the economic structure is in a stable “one-core, multiple sub-center” state, and the spatial polarization of core cities is still increasing. It verifies that China’s economic development has entered a new normal, the spatial polarization difference of economic development caused by the economic structure is gradually expanding, and structural adjustment and stable growth is the main goal of economic development in the Yangtze River Delta at the current stage.
(3) From the evolutionary relationship of spatial polarization, it is found that most cities are in a relatively stable spatial state without obvious transition, and the spatial polarization pattern of economic development has the characteristics of spatial locking. The spatial cohesion of the YRD region is strong, the spatially positive cities of economic development are concentrated in the YRD urban agglomeration, and the neighborhood relationship between cities is dominated by positive synergistic growth, while negatively polarized cities are mostly distributed in the negative neighborhood relationship. For the underdeveloped areas in the periphery of the YRD with coordinated growth, the spatial positive polarization effect of the economic quantity dimension and the spatial negative polarization effect of the economic structure dimension in the economic development of the YRD region are more significant.
(4) The degree of market prosperity and the urbanization level are both in the top three influencing factors in different research periods. The degree of marketization and urbanization in the YRD region is at a relatively high level in China, which plays an important role in reducing the regional economic spatial polarization level. In recent years, as the government of the YRD region vigorously promotes scientific and technological innovation, it has a significant driving effect on the reduction in the economic spatial polarization level, while the degree of openness and transportation convenience have relatively little impact on economic polarization. In the future, the steady improvement of marketization, urbanization, and science technicalization in the YRD region will contribute to the balanced and orderly development of cities and regional economies.

However, the following aspects still need to be further explored: (1) the basis of the economic spatial polarization studies, the agglomeration characteristics of urban land, culture, and technology, and the evolution process of spatial polarization are worthy of measurement and research. (2) The research scale can be further expanded from prefecture-level cities to county-level cities, depict the spatial polarization differences among counties and cities within the urban agglomeration, and explore the scale difference pattern of spatial polarization. It is the key direction to put forward more targeted policy suggestions for the coordinated development of the region.

## Figures and Tables

**Table 1:** Evaluation Index System of Economic Polarization Level.

First-Level Index	Secondary Index	Index Meaning
Economic quantity	Per capita GDP	Reflect on the overall level of economic development
	GDP growth rate	Reflect the rate of economic growth
Economic quality	labor productivity	Reflect the efficiency of labor production
	Per capita retail sales of consumer goods	Reflect the market consumption power
Economic structure	The added value of tertiary industry/added value of secondary industry	An index reflecting the upgrading of industrial structure
	Actual utilization of foreign capital per capita	Reflect the intensity of foreign investment

**Table 2:** Local Moran’s *I* transition probability matrix of economic spatial polarization in the YRD.

Period	*t*/*t* + 1	*HH*	*LH*	*LL*	*HL*	Type	Quantity	Proportion	*SF*	*SC*
2000—2019	***HH***	0.8735	0.0711	0.0237	0.0316	Type0	739	0.808	0.152	0.824
	***LH***	0.1497	0.7143	0.0816	0.0544	Type1	83	0.091		
	***LL***	0.0223	0.0471	0.8809	0.0496	Type2	56	0.061		
	***HL***	0.1518	0.1250	0.2054	0.5179	Type3	37	0.040		

**Table 3:** Selection of characteristic variables of the influence of economic polarization.

Dimension	Characteristic Variable	Indicator Description
Industrialization	Industrialization level	The proportion of industrial added to GDP
Urbanization	Urbanization level	The proportion of the urban population in the total urban population
Accessibility	Traffic convenience	The proportion of urban road ^1^ area to an administrative area
Openness	The opening degree to the outside world	The proportion of actual utilization of foreign capital in GDP
Marketization	Market prosperity	The proportion of total retail sales of consumer goods to GDP
Science technicalization	Technology level	The proportion of science expenditure in GDP

^1^ Urban road area refers to the urban pavement area and the area of squares, bridges, tunnels and sidewalks connected to the road. The sidewalk area is calculated by adding the areas on both sides of the road, including pedestrian streets and squares, excluding roads with mixed pedestrian and vehicle traffic.

**Table 4:** The detection results of factors affecting economic spatial polarization.

Characteristic Variable	2000			2009			2019		
	*q*-Value	*p*-Value	*q*-Value Sort	*q*-Value	*p*-Value	*q*-Value Sort	*q*-Value	*p*-Value	*q*-Value Sort
Industrialization level	0.492	0.000	2	0.363	0.004	4	0.404	0.000	4
Urbanization level	0.310	0.005	3	0.404	0.000	3	0.697	0.000	1
Traffic convenience	0.284	0.008	5	0.530	0.000	2	0.354	0.003	5
The opening degree to the outside world	0.285	0.008	4	0.229	0.010	6	0.197	0.016	6
Market prosperity	0.555	0.000	1	0.675	0.000	1	0.477	0.000	3
Scientific and technological level	0.249	0.009	6	0.302	0.008	5	0.589	0.000	2

## Data Availability

The data presented in this study are available on request from the corresponding author.
